# World Health Organization and knowledge translation in maternal, newborn, child and adolescent health and nutrition

**DOI:** 10.1136/archdischild-2021-323102

**Published:** 2021-12-30

**Authors:** STAGE (Strategic Technical Advisory Group of Experts), Trevor Duke, Fadia S AlBuhairan, Koki Agarwal, Narendra K Arora, Sabaratnam Arulkumaran, Zulfiqar A Bhutta, Fred Binka, Arachu Castro, Mariam Claeson, Blami Dao, Gary L Darmstadt, Mike English, Fadi Jardali, Michael Merson, Rashida A Ferrand, Alma Golden, Michael H Golden, Caroline Homer, Fyezah Jehan, Caroline W Kabiru, Betty Kirkwood, Joy E Lawn, Song Li, George C Patton, Marie Ruel, Jane Sandall, Harshpal Singh Sachdev, Mark Tomlinson, Peter Waiswa, Dilys Walker, Stanley Zlotkin

**Affiliations:** 1 Intensive Care Unit and University of Melbourne Department of Paediatrics, Royal Children’s Hospital, Parkville, Victoria, Australia; 2 Child Health, School of Medicine and Health Sciences, University of Papua New Guinea, Port Moresby, NCD, Papua New Guinea; 3 Leadership, Learning, and Development, Health Sector Transformation Program, Riyadh, Saudi Arabia; 4 College of Medicine, Alfaisal University, Riyadh, Saudi Arabia; 5 USAID Maternal Child Survival Program, Washington, District of Columbia, USA; 6 The INCLEN Trust International, New Delhi, India; 7 Obstetrics and Gynaecology, St George’s University of London, London, UK; 8 Centre for Global Child Health, The Hospital for Sick Children, Toronto, Ontario, Canada; 9 Institute for Global Health and Development, Aga Khan University, Karachi, Pakistan; 10 University of Health and Allied Sciences (UHAS), Ho, Ghana; 11 Department of International Health and Sustainable Development, Tulane University School of Public Health and Tropical Medicine, New Orleans, Louisiana, USA; 12 Department of Global Health, Karolinska Institute, Stockholm, Sweden; 13 Western and Central Africa, Jhpiego, Ouagadougou, Burkina Faso; 14 Department of Pediatrics, Stanford University School of Medicine, Stanford, California, USA; 15 Kemri-Wellcome Trust, Nairobi, Kenya; 16 Oxford University, Oxford, UK; 17 American University of Beirut, Beirut, Lebanon; 18 Duke Global Health Institute, Duke University, Durham, North Carolina, USA; 19 Clinical Research Department, London School of Hygiene and Tropical Medicine, London, UK; 20 US Agency for International Development, Washington, District of Columbia, USA; 21 Downings Letterkenny, Donegal, Ireland; 22 Burnet Institute, Melbourne, Victoria, Australia; 23 Pediatrics, Aga Khan University, Karachi, Sindh, Pakistan; 24 Population Dynamics and Sexual and Reproductive Health and Rights Unit, African Population and Health Research Center, Nairobi, Kenya; 25 London School of Hygiene & Tropical Medicine, London, UK; 26 MARCH Centre, London School of Hygiene and Tropical Medicine Faculty of Epidemiology and Population Health, London, UK; 27 National Health Commission of the People’s Republic of China, Beijing, China; 28 Adolescent Health, Murdoch Children’s Research Institute and The University of Melbourne, Melbourne, Victoria, Australia; 29 International Food Policy Research Institute, Washington, District of Columbia, USA; 30 Department of Women and Children’s Health, School of Life Course and Population Sciences, King’s College, London, UK; 31 Pediatrics and Clinical Epidemiology, Sitaram Bhartia Institute of Science and Research, B-16 Qutab Institutional Area, New Delhi, India; 32 Institute for Life Course Health Research, Department of Global Health, Stellenbosch University, Cape Town, South Africa; 33 School of Nursing and Midwifery, Queens University, Belfast, UK; 34 Makerere University, Kampala, Uganda; 35 Department of Obstetrics, Gynecology and Reproductive Sciences, Institute for Global Health Sciences, University of California San Francisco, San Francisco, California, USA; 36 Centre for Global Child Health, Hospital for Sick Children, Toronto, Ontario, Canada; 37 Department of Pediatrics, Faculty of Medicine, University of Toronto, Toronto, Ontario, Canada

## Abstract

The World Health Organization (WHO) has a mandate to promote maternal and child health and welfare through support to governments in the form of technical assistance, standards, epidemiological and statistical services, promoting teaching and training of healthcare professionals and providing direct aid in emergencies. The Strategic and Technical Advisory Group of Experts (STAGE) for maternal, newborn, child and adolescent health and nutrition (MNCAHN) was established in 2020 to advise the Director-General of WHO on issues relating to MNCAHN. STAGE comprises individuals from multiple low-income and middle-income and high-income countries, has representatives from many professional disciplines and with diverse experience and interests. Progress in MNCAHN requires improvements in quality of services, equity of access and the evolution of services as technical guidance, community needs and epidemiology changes. Knowledge translation of WHO guidance and other guidelines is an important part of this. Countries need effective and responsive structures for adaptation and implementation of evidence-based interventions, strategies to improve guideline uptake, education and training and mechanisms to monitor quality and safety. This paper summarises STAGE’s recommendations on how to improve knowledge translation in MNCAHN. They include support for national and regional technical advisory groups and subnational committees that coordinate maternal and child health; support for national plans for MNCAHN and their implementation and monitoring; the production of a small number of consolidated MNCAHN guidelines to promote integrated and holistic care; education and quality improvement strategies to support guidelines uptake; monitoring of gaps in knowledge translation and operational research in MNCAHN.

## Introduction

To achieve the Sustainable Development Goals (SDG), maternal, newborn, child and adolescent health and nutrition (MNCAHN) services must improve in quality and in equity of access. The World Health Organization (WHO) has a mandate to support governments to strengthening health services and improve public health, by providing technical assistance, epidemiological and statistical services, by promoting teaching and training of healthcare professionals, standards and by providing direct aid in emergencies. WHO has a specific mandate to promote maternal and child health and welfare.^1^

A common thread in improving health services is knowledge translation. WHO defines knowledge translation as ‘the synthesis, exchange and application of knowledge by relevant stakeholders to accelerate the benefits of global and local innovation in strengthening health systems and improving people’s health’.^2^ A working outline of knowledge translation is offered in box 1.^3 4^ Knowledge translation is a two-way iterative process, requiring engagement and learning with policy-makers, national and subnational governments and health managers, healthcare workers, families and the community and sectors outside health that are crucial to the health of mothers, children and adolescents. Knowledge translation should use existing experience and evidence, explore new technologies and maintain and build on knowledge of past successes and failures.

The Strategic and Technical Advisory Group of Experts (STAGE) for MNCAHN was established in 2020 to advise the director-general of WHO on issues relating to MNCAHN. STAGE comprises people from multiple low-income and middle-income countries (LMIC) and high-income countries, has representatives from many professional disciplines and with diverse experience and interests.^5^ Between April and October 2020, STAGE’s Knowledge Translation Working Group held four virtual meetings, the results of which were presented to the whole STAGE group for discussion and refinement. The findings were documented in a report to WHO in November 2020 and further refined in 2021. This paper, which summarises the report, proposes areas that WHO and national governments can act on to improve knowledge translation and implementation of MNCAHN programmes.

## Challenges In Translation Of Who Technical And Programme Guidance

For the implementation of WHO’s technical guidance and improving quality of MNCAHN care, there are several challenges:

▸ *Number and complexity of guidelines*: Countries receive a significant number of technical guidelines on individual diseases and interventions, from WHO and other sources. Strategies change quickly, leaving many countries behind on the uptake of updated information. This piecemeal approach can be confusing for healthcare workers and programme managers, and the guidance development process often appears non-transparent in relation to prioritisation of topics or directed by donors. Instead, the process should be more country-driven and region-driven, and ministries of health should be able to choose and prioritise guidelines and standards of relevance and adapt them to their context. Consolidated guidelines that are integrated may be more useful to healthcare practitioners than single disease and single intervention guidelines.
▸ *Limited resources at a country level for knowledge translation and dissemination*: Resources to determine policy, adapt technical guidance and operational tools and provide training are often limited. Consequently, new guidelines are slow to reach healthcare workers, managers and the other people for whom they are intended. There are deficits in sharing WHO guidelines and evidence in appropriate forms for preservice and in-service education of healthcare workers; integration of WHO guidelines within courses offered by colleges and schools that train healthcare worker is often slow or not done at all.
▸ *Health system constraints*: Health systems limitations make guideline implementation challenging, with gaps and competing priorities that must be simultaneously addressed. These include inadequate numbers, rapid turnover and inequitable distribution of healthcare workers; irregular supplies of drugs, equipment and other commodities needed to implement guidelines; lack of mentoring, supervision and continuing professional development programmes for healthcare workers. In addition, there are limited auditing or quality improvement processes to monitor guideline uptake, adherence and programme effectiveness.
▸ *Limited community engagement and communication*: Communication to healthcare workers and the community should be in appropriate languages and styles and use accessible media and new technologies; this is a lesson from COVID-19 in many countries.^6^ Communication strategies are best designed at country level and WHO has a role in supporting this. However, until recently WHO’s social media presence has been mostly high-level communication; videos were mostly in English and many of those on the WHO YouTube channel have been press conferences by senior WHO officials.^7^ Understanding the media that is the most common source of news and information accessed by local healthcare workers and the community is essential.
▸ *Limited engagement of non-health sector actors*: Engagement of government and non-governmental stakeholders outside the health sector—education, agriculture and food systems, social protection, finance, community development and urban planning—is important for the implementation of health programmes aimed at addressing the social, environmental and economic determinants within the SDGs.
  WHO recognises its key role in knowledge translation and the need to build more efficient processes to review data and revise recommendations, and to create guidelines and tools that are accessible to healthcare workers. WHO has made recent progress in these areas: some new WHO guidelines are digital and modifiable, which prepares the pathway for them to be incorporated into country’s digital platforms. For example, WHO has developed a specific toolkit for guideline adaptation and trialled this in relation to antenatal care guidelines.^8^ WHO is establishing practice networks to support peer learning and exchange, and has introduced the living guidelines concept, which facilitates rapid updating when new evidence become available.^9^ Although many of these initiatives are embryonic and need consistent support, leadership and advocacy, the newly developed WHO Academy, an online learning platform, will hopefully help and facilitate more engagement on healthcare worker training and continuing professional education.^10^
  In response to these challenges, STAGE makes three broad recommendations.

## Recommendation 1: Strategies To Improve Coordination And Oversight, The Importance Of Technical Advisory Groups And Subnational Committees

At a national and subnational level, many countries have committees that oversee policy in maternal and child health. These may be an overarching MNCAHN technical advisory group or committee (TAG), or several committees each with a focused remit: such as for maternal and newborn health; child health and nutrition and immunisation advisory committees. Such groups have a very important role. They have often evolved based on committment of individuals or professional associations, but reflect the limited resources available for MNCAHN, and many have minimal statutory endorsement and resources. Subnational MNCAHN committees, led by subnational health authorities (states, provinces or districts depending on the political structures) are often the drivers of implementation in devolved states and closer to where health services are delivered.

Based on these experiences, STAGE recommends that WHO and other partners should support ministries of health to strengthen these national and subnational advisory groups and build on the structures that already exist, and establish a global or regional resource centre for maternal and child health, such as with the national immunisation TAGs.^11^

WHO can play a normative, technical, enabling and capacity building role in the functions of such TAGs, and can support communication between committees. WHO has a role in ensuring external partners recognise the authority of such national and local committees, which can foster alignment and respect for national autonomy.

The criteria and terms-of-reference below provide general principles and are indicative only; local needs will dictate local approaches. It is important that TAGs be established within national regulatory structures to support the credibility of TAG recommendations and their accountability to national governments.

*National MNCAHN TAGs* can be the peak technical and monitoring bodies for MNCAHN in a country. They provide advice to the management or key personnel of the Ministry of Health or directly to executive government, and they provide policy and practice updates and recommendations to health managers, healthcare workers and other stakeholders. They are accountable to the Ministry of Health which sets the terms-of-reference but provide independent advice. The set-up and functions may include the following:

▸ Endorsement from the national government as a ‘statutory or standing body’, existing for the long term, with properly defined terms of reference and governance.
▸ Develop a comprehensive plan for MNCAHN that can act as a blueprint for national, subnational and local levels. Such a plan can guide annual implementation plans and inform programme managers, healthcare workers, the community and the government’s partners about maternal, child and adolescent health priorities and the approaches being adopted. The plan can be informed by WHO’s Redesign process.^12^
▸ Review and oversee the collection of essential primary data on MNCAHN and use this to guide policy and recommendations.
▸ Support capacity strengthening for monitoring and evaluation, data synthesis and implementation research.
▸ Provide leadership on adoption, adaptation and dissemination of evidence-based guidelines.
▸ Initiate and oversee a MNCAHN Quality Improvement Programme. Such a programme may cover all aspects of quality improvement, including health facility accreditation, education and continuing professional development, standards and assessment, audit, small group problem solving, communication of local initiatives in order to improve quality of care. This could include supporting ‘Centres of Excellence in MNCAHN’, including health facilities at a district or subnational level.
▸ Oversee an annual ‘State of the Nation’s Mothers, Newborns, Children and Adolescents Health’ report that brings together data on health, nutrition, education and other SDG targets. This would be an important monitoring and evaluation exercise leading to follow-up and actions at national and subnational level, and to increasing accountability.
▸ Strategic thinking at country level on how often to update guidance and how to do it: from ‘simple’ changes (eg, substitution of one drug for another) to more complex changes (eg, shifting a task from one cadre to another).
▸ Advocate for adequate government budget allocation, funding and resource mobilisation for guidelines and recommendations to be implemented.
  The functions that a national TAG takes on will depend on its existing capacity but WHO should be able to help countries to grow these capacities, and support resource mobilisation across partners.

## Membership and links

The membership of the national TAG should be determined in-country. It should ensure intellectual independence, lack of conflict of interest, allow for a range of perspectives and support the alignment and dissemination of key decisions. Members could include personnel skilled in MNCAHN epidemiology, disease burdens and health systems, professional associations, key academic/university personnel, subnational representatives, UN agencies (WHO and UNICEF) and sectors beyond health, such as education, finance and law, and the private sector where relevant. Membership should also include consumer, community and civil society representatives, such as women’s groups, a community youth leader and Indigenous groups where they exist. Membership should also include frontline healthcare workers who will be tasked with implementing the guidance and policies, such as a midwife, child health nurse or allied healthcare professional.

*Subnational* (state, province or district) *MNCAHN committees* are needed to operationalise recommendations and the national plan for MNCAHN in countries with devolved systems, to contextualise it to local priorities and to oversee local operations and monitoring. Subnational or local committees should have wide representation, including frontline healthcare workers such as a midwife, child health nurse or allied healthcare professional, and ensure that civil society has a voice in decision making: such as a community leader, women’s group or representatives of other sectors such as a teacher.

*Regional TAGs for MNCAHN* would provide WHO regional directors and countries in the respective regions with MNCAHN strategic priorities and technical recommendations considering new global guidance and its regional relevance. Regional TAGs would support regional exchange and national capacity building. The regional TAG structure and procedures would reflect the needs and capacities of its member states. Links between national and regional TAGS are important. One way to enable this may be that national TAG chairs are represented on the regional TAG.

## Recommendation 2: Strategies To Improve Guideline Uptake

STAGE recommends that WHO:

▸ *Produce a small number of consolidated MNCAHN guidelines to promote integrated and holistic care* Examples include the Pocketbook of Hospital Care for Children,^13^ the Pocketbook of Hospital Care for Mothers, in the South East Asian Region (SEARO) of WHO^14^ and the Pocketbook of Primary Care for Children in the European Region of WHO (EURO). Such consolidated guidelines are more useful to healthcare practitioners than multiple individual single disease guidelines. These consolidated guidelines would be regularly updated as new evidence becomes available, and consistently supported. They would strengthen long-term incorporation of WHO guidelines into the health system culture. Consolidated guidelines should be easily adaptable to promote ownership, for example, to enable co-branding by national ministries of health or professional associations. They may be global, as in the first example above, that can be adapted regionally or nationally, or regional as in the latter two examples. They may be in digital form, in addition to the traditional guideline handbooks. In the development of such resources, the input of frontline workers should be sought, as they will be asked to implement such guidance. There should be education and training resources linked to guidelines, for use by health services, ministries of health and schools and colleges of healthcare worker training for preservice education and continuing professional development.
▸ *Develop a comprehensive operational handbook for MNCAHN that provides programmatic and training guidance for implementation which can be adapted and owned at national level*: Such a handbook could include guideline adaptation tools, programmatic advice, decision-making tools for frontline staff, training aides and recommendations on management, training, supervision, monitoring and evaluation, integration of services, quality improvement and implementation research.
▸ *Support guidelines produced or adapted by national ministries of health and national healthcare professional associations*: While other international non-government organisations (NGOs) and UN agencies may also develop guidelines, those developed nationally and locally should be supported. National healthcare professional associations are the most important group to support for implementation, ideally brought together (paediatricians, obstetricians, nurses, midwives and allied health). WHO can make their guidelines more easily adaptable with provision for co-branding, and in formats that are modifiable.
▸ *Encourage and support national ministry of health guideline websites*: to house locally adopted and endorsed guidelines and operational handbooks. Many ministries of health in LMIC have rudimentary and outdated websites, so improving digital capacity and infrastructure to address this local need is important. Work is also needed to support digital platforms for mobile phone and other app-related approaches to find ways to enable healthcare workers to have easy access to the guidance. Ministries of health should have a specific person responsible for keeping track of new guidelines coming out across MNCAHN.
▸ *Support National MCH Quality Improvement Programmes*: A quality improvement programme may be multifaceted, including health facility accreditation, healthcare worker education and continuing professional development, standards and assessment, audit, small group problem solving, communication of local initiatives, in order to address the health system bottlenecks in improving quality of care. It is not WHO’s role to initiate such a national programme, but technical support and endorsement will be invaluable. There may be quality improvement programmes in other areas (eg, HIV, immunisation), where there could be synergies and lessons shared.
▸ *Develop a new WHO programme of support to institutions of healthcare worker training in LMIC*: to increase the teaching of WHO guidelines and address healthcare worker deficiency. In many low-income settings, colleges of nursing, midwifery, medical, allied health training are underfunded and under-resourced, and output is inadequate to meet demands. This stifles progress in all the health-related SDG targets. The inequities in healthcare worker numbers, distribution and training and the tragic consequences have been starkly highlighted by the COVID-19 pandemic, which risks leaving a seismic gap in healthcare worker numbers in the coming decade. A WHO programme of support to schools and colleges of healthcare worker training could lead to greater incorporation of WHO guidelines into curricula, increased capacity of educators, argue for more funding for healthcare worker training institutions through global projects and local budgets, facilitate links with other organisations that would support such institutions, including accreditation bodies and produce curricula for nursing and other healthcare professional training that could be adapted locally. New online teaching methods and the WHO Academy will play a role.^10^ It will be important to pair this with standards and certification for continuing professional education and the professional accreditation body keep track and maintain accountability.
▸ *Develop child health nurse training as a postgraduate course supported by WHO*: in the same way that WHO and other agencies have promoted midwifery training globally. Child, neonatal and adolescent health is far more complex in the SDG era; there is so much more to be learnt than can be taught in preservice general nursing courses. A generic curricula could be developed, based around WHO guidelines, which would bring together all the relevant guidelines and consolidate the many ‘short-courses’ into a 1-year to 2-year practical postgraduate course (primary child healthcare, nutrition, hospital care, newborn care, HIV, tuberculosis, immunisation, adolescent health, care of children with chronic conditions, child protection, disability, quality improvement). It would encompass prevention and treatment and teach principles of family centred care and equity. Such a course would be open to general nurses, nurses with experience in paediatrics, midwives and even non-medical clinical officers working in child health, for which there are often no ongoing career pathways. Many countries need an accreditation process for any new course and healthcare worker credentialing.^15 16^
▸ *Develop more multimedia outputs including videos in multiple languages*: WHO should explore capacity to be more creative, engaging and multilingual in its communication using this medium to communicate science-based public health information. More videos could target an audience that includes healthcare workers in the field, families and communities. This would take resources: skilled people and time, helped by an adolescent understanding of social media sites. WHO-endorsed YouTube or other social media clips could also tell local stories of successful implementation—even encourage end-users to make videos of their own lessons learnt; these could be developed at a local or national level, and reviewed or endorsed by WHO if suitable. WHO may also develop or endorse digital mobile apps that are linked to WHO guidelines.
  Responsibility for funding is mixed. WHO has the responsibility to fund global guideline development, countries have responsibility to fund implementation, with support from diverse partners. Funding or in-kind support for implementation of a national maternal, newborn, child and adolescent health plan is typically provided by a consortium of government, international organisations, local NGOs and voluntary organisations. The make-up of the national and regional committees includes individuals who are already paid by the government or local university, with some resources from WHO, governments or partners to ensure equity of representation.

## Recommendation 3: Monitor Implementation Of Mncahn Care And Gaps In Knowledge Translation At A National And Local Level

Countries need well-functioning monitoring processes to identify the gaps in implementing recommendations for MNCAHN. TAGs have a role in reviewing data, including health system implementation data, and routinely collected health activity and outcome data. Data should reflect problems faced by those who directly manage and deliver services in the field. With a focus on equity, monitoring interventions and health outcomes in disadvantaged communities is essential to measure fair access and universal health coverage. Human resource, education and training data need also be included. While many metrics have been proposed, countries have autonomy to decide on indicators and methods of measurement that are feasible, meaningful, valid, and sustainable.^17^

There is value in reporting where implementation of MNCAHN guidelines and models of integrated care have been successful. This could include exemplar case studies of models of care or guideline implementation that are clearly articulated and explore objectively the elements of that success and the challenges. Policy makers, managers and clinicians would benefit from such examples. Sharing of lessons supports continuous long-term learning and enables a memory and cumulative strengthening of what works.

## Conclusion

Adaptation and implementation of WHO and other guidelines is necessary to improve the quality and fair access to MNCAHN services, towards achievement of universal health coverage and the SDGs, and building back better after the pandemic. Countries need effective and responsive structures to improve guideline uptake, training and continuing professional education for maternal and child healthcare workers, and mechanisms to monitor quality and safety. A governance structure with a technical advisory committee, a unified national plan that acts as a blueprint for progress and a feasible monitoring strategy are the building blocks. WHO has an important role in supporting these processes at global, regional, national and local levels, and remains a trusted collaborator for MNCAHN knowledge translation.

## Data Availability

Data are available in a public, open access repository.

## Box 1 Knowledge translation components and processes

Knowledge synthesis (including analysis of research, guideline or policy development, investment cases, development of user-friendly guidance).
⇒ Dialogue/exchange (deliberative dialogue for guideline development, prioritisation, budget allocation and adoption strategies).
⇒ Adaptation to context so that policies and guidelines take account of local epidemiology, feasibility, affordability, social and cultural values, equity and preferences - that they are fit for purpose in the environments where most needed.
⇒ Evidence-informed programme design, monitoring and evaluation.
⇒ Activities where knowledge, guidelines or recommendations are used in the provision of healthcare or other services.
⇒ Appropriate training and incorporation of new guidelines into the local health culture and health education.
⇒ Behaviour change to enhance the use of guidelines and recommendations in ways that improve health at an individual and population level.
⇒ Evaluation of reach, uptake, acceptability and effectiveness.

## What is already known on this topic?

⇒ WHO produces numerous guidelines on aspects of maternal, newborn, child and adolescent health, and countries have difficulty with implementation, in part because resources for knowledge translation are limited.
⇒ There are many health system constraints to implementation of WHO guidelines, including inadequate numbers, inequitable distribution and lack of support for healthcare workers.
⇒ The COVID-19 pandemic is leaving maternal, newborn, child and adolescent health and nutrition (MNCAHN) services depleted in many countries, and there is a need to maintain focus on improving quality and equity of access to services.

## What this study adds?

Proposes several steps for the translation of knowledge in maternal, newborn, child and adolescent health and nutrition.
⇒ Proposes a structure for implementation of MNCAHN programmes and quality improvement across regions, and within countries.
⇒ Emphasises the need to focus on healthcare workers, local needs and strengthening capacity.
